# Brachiocephalic Artery Dissection Following Type A Aortic Dissection Repair

**DOI:** 10.7759/cureus.51379

**Published:** 2023-12-31

**Authors:** Nashwan Alattab, Shatha A Althobaiti, Nasser S Alwehaibi, Saleh T Mahjoub

**Affiliations:** 1 Vascular Surgery, King Fahad Medical City, Riyadh, SAU; 2 General Surgery, Al-Noor Specialist Hospital, Makkah, SAU; 3 Vascular Surgery, King Saud University, Riyadh, SAU; 4 College of Medicine, King Saud University Medical City, Riyadh, SAU

**Keywords:** case report, aortic dissection, type a aortic dissection, endovascular intervention, bca dissection

## Abstract

Acute type A aortic dissection (ATAAD) is a life-threatening emergency that is associated with major morbidity and mortality. Arterial dissections, particularly the brachiocephalic artery, can remain as a residual dissection after type A aortic dissection repair. We present a rare case of brachiocephalic artery dissection due to the clamping effect and the management of ATAAD patients. A 47-year-old male known for aortic aneurysm and uncontrolled hypertension presented with high blood pressure, unequal pulses, and a history of chest pain. A thoracic and abdominal aorta angiogram showed aneurysmal dilatation of the aortic root and ascending aorta with a peripheral linear filling defect shortly distal to the aortic root. The patient underwent the Bentall procedure, hemi-arch replacement, and patent ductus arteriosus closure. The brachiocephalic artery was clamped. The angiogram showed right common carotid occlusion. Endovascular intervention was made by balloon-mounted covered stent graft and kissing technique. The patient had a smooth post-procedure period without major events. Iatrogenic brachiocephalic artery dissection can occur during type A aortic dissection repair and is frequently affected by residual dissection. The decision of intervention versus conservative management is based on a patient's general condition.

## Introduction

Arterial dissections and damage, particularly the brachiocephalic artery (BCA), can result from blunt trauma or remain as a residual dissection after type A aortic dissection repair [1,2]. The most widely and currently used technique for the management of acute type A aortic dissection (ATAAD) consists of the replacement of the ascending aorta and “Hemiarch” with an open distal anastomosis, with either aortic valve resuspension and obliteration of the aortic root false lumen or a Bentall’s procedure [3]. Imaging investigations are indicated following Bentall’s procedure for the evaluation of suspected complications and for routine follow-up in asymptomatic patients; computed tomography (CT) is the modality of choice [4]. Here, we discuss a rare case of BCA dissection due to the clamping effect and management of ATAAD patients.

## Case presentation

A 47-year-old male patient with an aortic aneurysm and uncontrolled hypertension presented to the emergency department with a history of chest pain. At the presentation, his blood pressure (BP) was around 200 mmHg with unequal pulses in the upper limbs, and his right arm systolic BP of 30 mmHg was less than the left limb.

CT angiogram of the thoracic and abdominal aorta showed aneurysmal dilatation of the aortic root and ascending aorta reaching 6.2 cm with a peripheral linear filling defect in the ascending aorta shortly distal to the aortic root. The descending thoracic aorta was dilated and measured 3.2 cm. Contrast-filled communication was noted between the pulmonary trunk and the aortic arch suggestive of patent ductus arteriosus. The dilated pulmonary trunk measured 4 cm.

The patient was shifted to an operative room and underwent Bentall’s procedure, hemiarch replacement, and patent ductus arteriosus closure done by a cardiac surgeon. Intra-operatively, dissection was very limited to the anterolateral aspect of the aorta, BCA was clamped, ante-grade cerebral perfusion was given through the right axillary artery, and there was no dissection. One day postoperatively in ICU and during the attempt of extubation, the patient was not moving his left side, stroke code was activated, and CT of the brain showed multiple ill-defined hypodense areas involving the right middle cerebral artery (MCA) territory with perfusion defect representing acute infarction. CT angiogram showed right common carotid occlusion. Neuro-intervention was contacted and the patient was shifted to the neuro-intervention lab. Selective angiography was done and showed dissection beyond the common carotid artery. By consulting a vascular surgeon and under general anesthesia, access was achieved through the right femoral artery to the BCA.

**Figure 1:**
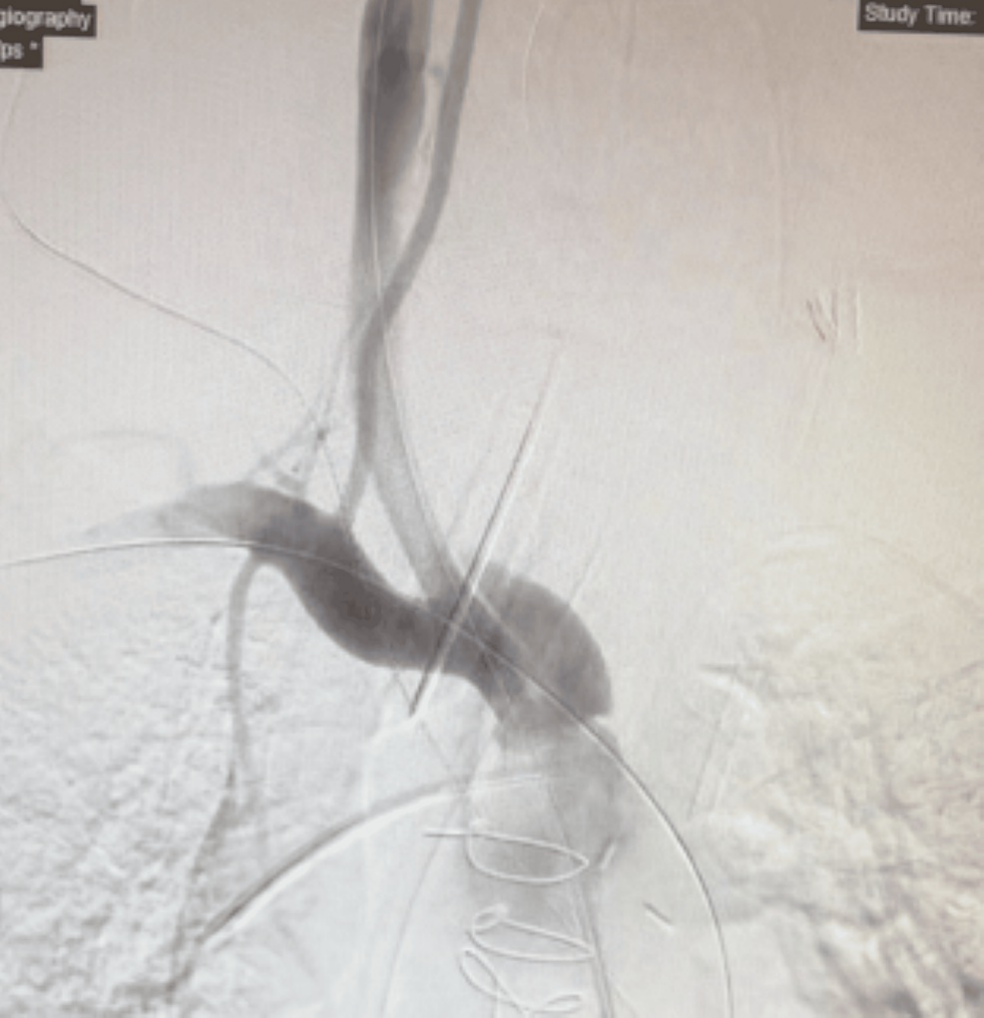
Dissection of the proximal brachiocephalic artery

The angiogram revealed a dissection of the right common carotid artery. The subclavian artery showed good flow distally. Injection of the right vertebral artery showed retrograde flow that is suggestive of a proximal occlusion. Flow-limiting dissection of the proximal BCA was noted (Figure 1).

**Figure 2:**
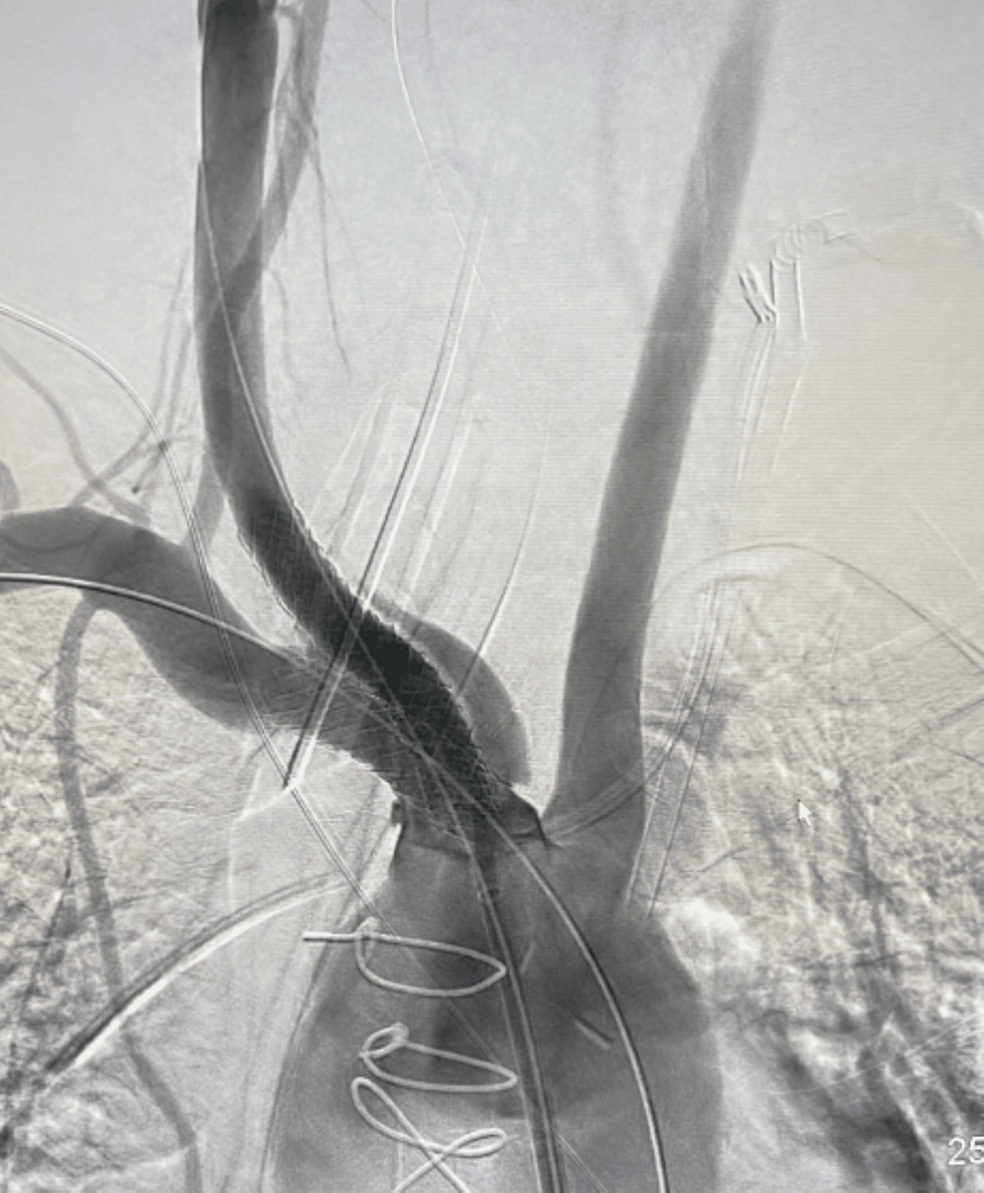
Stenting of the brachiocephalic artery, right common carotid artery, and right subclavian artery by balloon-mounted covered stent graft, and kissing technique crossing the dissection

The most likely suggestion was having spiral dissection starting at the proximal BCA into both the subclavian and right common carotid artery. Another access was made through the right brachial artery, stenting of the BCA, right common carotid artery, and right subclavian artery by balloon-mounted covered stent graft, and kissing technique simultaneously inflated crossing the dissection (Figure 2).

A selective angiogram of the right common carotid artery showed a good flow in the right common carotid, the bifurcation, and the intracranial vessels. An injection of the right subclavian artery showed a good antegrade flow of the right vertebral artery. The patient was sent back to the intensive care unit (ICU). The 8F sheath was removed by cutting down, followed by primary closure of the arteriotomy. One day postoperatively, a CT of the brain showed stable MCA acute stroke with no hemorrhagic transformation. The patient had a smooth post-procedure period without major events, and he was on regular physiotherapy. Before discharge, the evaluation of the patient showed that the power of the left upper limb was 0/5, and the lower left was 1-2/5. The right side was moving well and the patient started to walk; the power of the left upper limb was 3/5 and the left lower limb was 4/5 after six months of physical therapy.

## Discussion

BCAs are frequently affected by residual dissection in patients with type A aortic dissection [1]. In a study carried out among patients with spontaneous type A aortic dissection, they found that 42 of 137 patients with spontaneous aortic dissection were recognized as having residual dissection of the BCAs [1]. In our case, BCA dissection was most likely due to the clamping effect since throughout the first surgery dissection was very limited to the aorta. The BCA was clamped and there was no dissection intra-operatively. To our knowledge, no study addressed the incidence of iatrogenic BCA dissection. Despite that, surgical manipulation and trauma such as cannulation, clamping, or incisions initiate injury to the intima and pulsatile blood flow within the lumen that perpetuates the dissection [5]. A patient with BCA dissection may present with severe hypotension and neurological deficits [2]. Postoperative cerebral complications are frequent for patients with ATAAD [6]. In a study that included 202 patients with ATAAD who encountered surgical repair, it was found that only BCA dissection was a significant risk factor for stroke after ATAAD repair on multivariable analysis. Postoperative stroke was observed in 12%, 30-day mortality was 6%, whereas hospital mortality was 8% [7]. In another study on the residual dissection group of patients, it was concluded that the residual dissection of the BCAs exposed the patients to an increased risk of neurologic issues in the territories depending on the dissected arteries [1]. The gold standard in vascular imaging is diagnostic angiography. Rapidly non-invasive imaging modalities such as duplex arterial mapping, computed tomography angiography, and magnetic resonance angiography decrease its use [8]. Laura Seese and colleagues reported a case of acute dissection involving the origin of the right BCA that reached the right common carotid and right subclavian arteries. The patient was managed conservatively with a nonoperative management approach. Taking into consideration the isolated nature of this dissection and the lack of neurological symptoms and indication of aortic or innominate artery replacement, the patient was treated with a strict antihypertensive regimen with warfarin and aspirin to prevent additional thrombotic potentiation. In neurologically asymptomatic patients without indications for endovascular or operative intervention, conservative management with a strict antihypertensive medication, with or without antiplatelet therapy, and close follow-up for ischemic neurological sequelae may be an acceptable approach [9]. Indications for surgical intervention include having symptoms of carotid hypoperfusion, symptomatic subclavian steal syndrome, and multiple transient ischemic attacks (TIAs) despite antithrombotic medications or positive microembolic signal (MES) counts at transcranial Doppler (TCD) examinations [1]. Eugenio Neri and colleagues discussed some cases that were treated by the endovascular approach with no more surgical details [1]. The chosen method of open arterial repair depends on the extent and the mechanism of injury. Large injuries may require prosthetic grafts, whereas, for less complex injuries, a vascular patch, or performing lateral arteriography can be done [2]. Our patient developed a stroke; furthermore, he was hemodynamically unstable and intubated, so the decision was taken to do an endovascular repair as the open technique would increase morbidity and mortality in this situation. An open technique would be a choice if we failed to do it by endovascular technique.

## Conclusions

Iatrogenic BCA dissection can occur during type A aortic dissection repair and is frequently affected by residual dissection. It presents as neurological deficits and severe hypotension. CT angiogram confirms such complication. The decision of intervention versus conservative management is based on the patient's general condition. Our patient presented with severe symptoms after an iatrogenic injury. The surgeon should be aware of such acute complications and not delay the proper management.
